# Use of the Electrified Wire Technique for In Situ Fenestration Creation Within a Branch Bridging Stent

**DOI:** 10.1177/15266028251324074

**Published:** 2025-03-13

**Authors:** Antonino Giordano, Petroula Nana, Giuseppe Panuccio, Kugarajah Arulrajah, José I. Torrealba, Fiona Rohlffs, Tilo Kölbel

**Affiliations:** 1 German Aortic Center, Department of Vascular Medicine, University Heart and Vascular Center UKE Hamburg, Hamburg, Germany

**Keywords:** electrified wire technique, bifurcated bridging stent, bailout techniques, branch endovascular repair

## Abstract

**Purpose:**

To present the use of the electrified wire technique as bailout for target vessel (TV) branch preservation after unintended coverage by a bridging stent during branched endovascular repair (bEVAR).

**Technique:**

A 73-year-old male, previously treated with thoracic endovascular aortic repair and Provisional Extension to Induce Complete Attachment Technique (PETTICOAT) for type B aortic dissection, presented with a 68 mm type V thoracoabdominal aortic aneurysm. The patient presented an anatomic variation with a common trunk for the superior mesenteric artery (SMA) and celiac artery (CA). After a previously failed open surgical attempt, a triple-branch custom-made device (2 renal branches and 1 for the SMA/CA trunk) was chosen. Endograft deployment and TV catheterization were uneventful, until an unintended coverage of the CA occurred, due to bridging stent unmount. Using the electrified wire technique, an in situ fenestration was created into the CA/SMA trunk covered bridging stent to preserve CA patency. A bare metal stent was used for CA revascularization. Renal arteries were catheterized and bridged as planned. The 6-month computed tomography angiography confirmed TV patency.

**Conclusion:**

The electrified wire technique may be used as bailout for in situ fenestration creation in unintended coverage of early TV side-branches.

**Clinical Impact:**

Anatomic variations may increase the technical complexity of branched endovascular aortic repair while technical pitfalls related to target vessel preservation may demand the application of bailout techniques. In this case, the electrified wire technique was used to create an in situ fenestration within the bridging stent of a celiac artery (CA) and superior mesenteric artery common trunk (anatomic variation), after bridging stent unmount during advancement and CA unintended coverage.

## Introduction

Branched and fenestrated endovascular aortic repair (f/bEVAR) is applied in high-volume centers with high technical success rates.^1^ Distal sealing in target vessels (TVs) with bridging covered stents (BCS) can be challenging in case of early side branches, especially in patients presenting anatomic variations.^2,3^ Celiac artery (CA) coverage in fEVAR or absence of bridging in bEVAR may be decided in limited cases with preexisting severe CA stenosis and well-developed collateral flow.^4^ However, CA preservation should always be attempted to preserve collateral beds and avoid upper gastro-intestinal ischemia, especially in case of extended aortic coverage and pre-operative CA patency.^4^

Despite increasing experience, technical difficulties in TVs bridging may occur intraoperatively and an increasing number of bailout techniques has been applied to solve these challenges.^2,3^ In situ fenestration has been used for visceral vessel or supra-aortic trunk preservation, in urgent setting, using either mechanical means, such as re-entry catheters, or laser technology.^5,6^ The electrified wire technique is an additional low-cost, effective off-the-shelf solution that could be applied for fenestration creation, even within anatomic structures of limited diameter or tortuous morphology, where larger and stiffer material would lose navigability and trackability.^7^

This technical report describes the use of the electrified wire technique, as a bailout for in situ fenestration creation within a bridging stent covering a large early side-branch in a bEVAR patient.

## Technique

**Figure 1.**
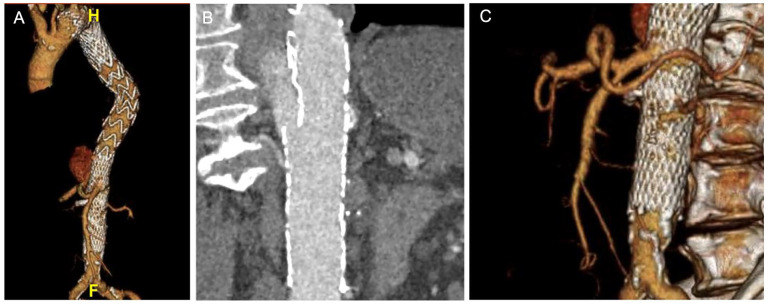
The preoperative computed tomography angiography confirmed the presence of a 68 mm type V thoracoabdominal aneurysm in a patient previously treated with thoracic endovascular repair and distal PETTICOAT for an acute complicated type B aortic dissection (Panel A). Potentially cause–effect relationship between aneurysm formation and bare metal stent fracture cannot be excluded (Panel B). The preoperative CTA also confirmed the presence of an anatomic variation with a common trunk for the celiac artery and superior mesenteric artery (Panel C). CTA, computed tomography angiography; PETTICOAT, Provisional Extension to Induce Complete Attachment Technique

A 73-year-old male presented with a 68 mm type V thoracoabdominal aneurysm (TAAA) according to modified Crawford classification.^8^ In 2018, the patient was diagnosed with aortic rupture due to acute type B aortic dissection and was treated with thoracic endovascular repair in another institution. Six months later, he received a Provisional Extension to Induce Complete Attachment Technique (PETTICOAT) and a stenting of the right renal artery (RRA), due to incomplete expansion of the true lumen and worsened renal function. For the PETTICOAT, a 36 mm Sinus-XL (Optimed, Ettlingen, Germany) stent was used. Afterward, the patient remained asymptomatic. In October 2022, a follow-up computed tomography angiography (CTA) revealed the presence of a type V TAAA of the false lumen and multiple bare metal stent fractures (Figure 1A and B). An endovascular approach was considered excessively complicated at another institution, and the patient was offered open surgical repair, which was attempted but stopped prematurely due to severe intraoperative bleeding.

**Figure 2.**
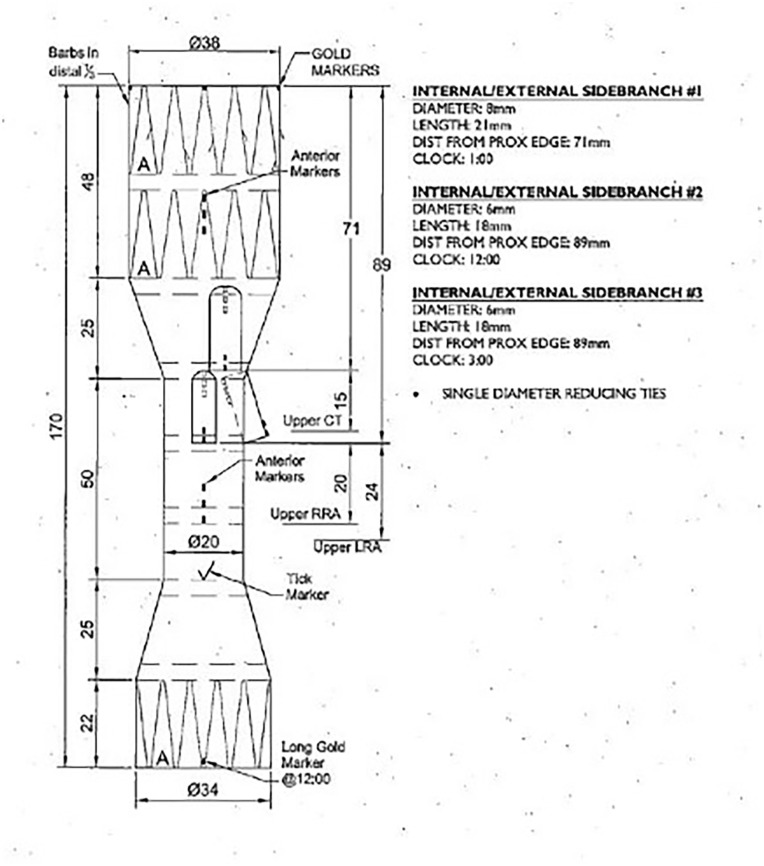
A custom-made branched was designed (Cook Medical), with 3 outer branches dedicated to the common celiacomesenteric trunk and the 2 renal arteries.

The patient was then referred to our center for further management. Despite technical challenges arising from the previous endovascular interventions and considering the presence of a hostile abdomen, an endovascular approach with a custom-made branched device was proposed (Cook Medical, Bloomington, IN, USA). The preoperative CTA confirmed the previously mentioned findings, in addition to the presence of an anatomic variation of the celiac artery (CA) and superior mesenteric artery, presenting with a common trunk [celiaco-mesenteric trunk (CMT); Figure 1C]. A custom-made device with 3 outer branches dedicated to the CMT, RRA, and left renal artery was designed (Figure 2).

## Technique

**Figure 3.**
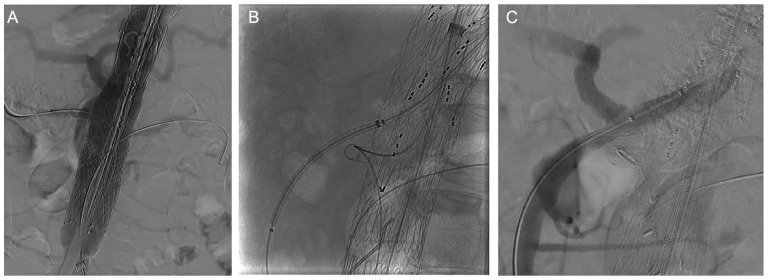
To ensure renal artery stenting, a precatheterization of both renal arteries was performed through the left femoral access, while the right femoral access was used for the introduction of the main device (Panel A). The device was deployed as scheduled and with the use of a steerable sheath, the celiacomesenteric trunk was catheterized first. An 8 × 57 mm, Advanta V12 (Atrium, NH, USA) was advanced into the vessel (Panel B). In order to perform a selective angiography, the stent was further advanced into the vessel and then, retracted. However, during retrieval, the stent partially slipped off the balloon it was crimped on, leading to a misdeployment and celiac artery occlusion (Panel C).

The patient was operated in supine position under general anesthesia in a hybrid operating theater. A cerebrospinal fluid drainage was placed preoperatively.^9^ Bilateral percutaneous common femoral artery (CFA) access with short 6 Fr sheaths were performed under ultrasonographic guidance. Systemic heparinization at 100 IU/kg with a target activated clotting time >250 seconds was obtained. Under fluoroscopic fusion guidance and to reassure renal artery stenting, a precatheterization of both renal arteries was performed through a 14 Fr Flexor (Cook Medical) sheath from the left CFA access. The right CFA access was used for the introduction of the main device (CMD 38-34-170; Cook Medical; Figure 3A).

The main device was deployed with the proximal branch 2 cm above the CMT and then, retrieved. Through the right CFA access, a 14 Fr sheath (Flexor; Cook Medical) was advanced, followed by a steerable sheath (10 Fr × 55 cm, Fustar; Lifetechmed, Shenzhen City, China) stabilized with a 0.014′ wire as previously described.^10^ Using a hydrophilic 0.035″ guidewire and a 100 cm 5 Fr Berenstein catheter, the branch and CMT were subsequently catheterized, followed by the placement of Rosen wire and a 7 Fr × 70 cm Flexor Sheath advancement into the TV. A balloon expandable covered stent (8 × 57 mm; Advanta V12) was chosen as BCS and advanced into the TV (Figure 3B). As the size of the sheath would not permit an appropriate angiography after stent advancement, the stent was further advanced into the TV, and the angiography was performed. The stent was then retracted into the appropriate position and expanded. However, during retrieval, the stent partially slipped off the balloon, it was crimped on (an incident that remained unnoticed due to the balloon markers being in the correct position and low visibility of the BCS itself in a lateral angulation), leading to an misdeployment and CA coverage (Figure 3C).

**Figure 4.**
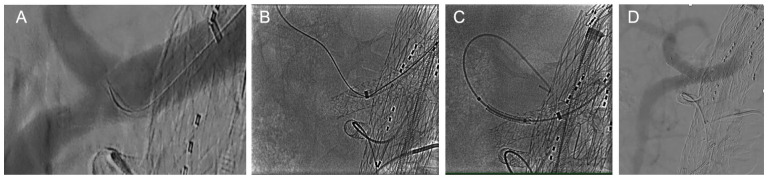
In order to revascularize the celiac artery, the electrified wire technique was used. A Vector 3 catheter was place into the stent facing almost at 90° the celiac axis ostium. The PTFE coating of a short proximal segment of a 0.014″ Astato (Astato XS; Asahi Intecc) wire was stripped off on table, and the wire was advanced into the catheter (Panel A). The electrocautery was activated, and in coordinated motion, the wire was pushed against the stent, creating the fenestration (Panel B). After full expansion of the branch bridging stent, balloon angioplasty of the fenestration was performed and an 8 × 17 mm^2^, Visi-Pro (Medtronic) was advanced, deployed, and flared (Panel C), to restore and preserve celiac artery patency (Panel D).

In order to revascularize the CA, the electrified wire technique was used. The setup has been reported previously.^11^ A Vector 3 catheter (Merit Medical, South Jordan, UT, USA) was placed into the stent facing the CA ostium. A Tuohy–Borst valve was attached to the catheter, and an infusion of 5% glucose was initiated. The Polytetrafluoroethylene (PTFE) coating of a short proximal segment of a 0.014″ Astato (Astato XS; Asahi Intecc, Amsterdam, Netherlands) wire was stripped off on-table, and a mosquito clamp was attached to ensure safe current passage. After modification, the wire was advanced into the catheter, and the catheter was positioned in close proximity to the stent, in a near 90° position (Figure 4A). The electrocautery was activated in cutting mode at 70 W, in contact with the bare Astato segment and, in a coordinated motion, the wire was pushed against the stent. After penetrating the wall of the covered stent, the wire was advanced into the CA (Figure 4B). Due to difficulty in balloon and catheter advancement, an 8 mm balloon was used to fully expand the BCS, thereby thinning the PTFE and further separating the stent-structures, and a second successful attempt was performed. The fenestration was initially dilated using coronary balloons (1.2 × 8 mm, Emerge Push; Boston Scientific, Voisin-le-Bretonneux, France and subsequently, 1.5 × 12 mm Sapphire 3; Orbusneich, Hoevelaken, Netherlands) and then, a 5.5 × 20 mm (Viatrac 14 Plus; Abbott, Inc., Des Planes, IL, USA ) peripheral angioplasty balloon. The CA bridging stent was advanced (8 × 17 mm, Visi-Pro; Medtronic, Santa Ana, CA, USA), deployed, and flared through the Advanta into the CA (Figure 4C). Another BCS (8 × 32 mm; Advanta V12 Atrium, NH, USA) was used to further connect the first CMT stent into the branch and a selective angiography showed stent patency, without endoleak (Figure 4D).

**Figure 5.**
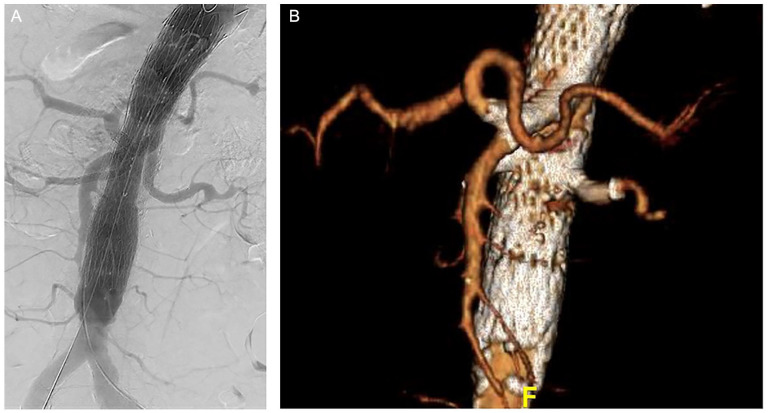
The completion angiography showed patent visceral vessels (Panel A), while the 6-month computed tomography angiography confirmed endograft and bridging stent patency, including the celiac artery fenestration (Panel B).

The catheterization and connection of the 2 renal arteries was uneventful, using the previously described steps. Both renal arteries were bridged with self-expanding covered stents (Viabahn; W. L. Gore & Associates, Inc., Flagstaff, AZ, USA). The completion angiography showed the appropriate position of the endograft and TV patency, in addition to a type IIb endoleak (Figure 5A). Sheaths and wires were retrieved, and CFAs were closed with Prostar XL device (Abbott) for the right side and Manta 14 Fr (Teleflex Medical, Athlone, Co Westmeath, Ireland) for the left side. The duration of the procedure was 320 minutes, with a total fluoroscopy time at 74 minutes and radiation dose at 5869.62 cGy cm^2^.

Postoperatively, the patient was monitored for 12 hours in the intensive care unit. The predischarge CTA confirmed endograft and TVs’ patency, as well as the type IIb endoleak. The postoperative course was uneventful, and the patient was discharged the eighth postoperative day, under double antithrombotic therapy (Apixaban 5 mg, twice daily due to known atrial fibrillation and clopidogrel 75 mg, once daily). The 6-month CTA confirmed once again the predischarge findings, without the presence of endoleak (Figure 5B).

## Discussion

bEVAR is a safe and reliable solution for TAAA repair while device customization permits its application in a wide target-population, with broad spectrum of anatomic characteristics.^1,12^ Challenging anatomies, like tortuous and angled TVs, narrow inner aortic diameters and previous endovascular repair may increase technical challenges and lead to pitfalls, as shown in the current case, where a stent misplacement occurred after the advancement of the device further into the TV during an angiography performance. In these cases, bail-out techniques are a useful tool to overcome complications and provide life-saving solutions.^8^ The electrified wire technique is a low-cost off-the-shelf representative of them.^7^ In the current case, the creation of an in situ fenestration into a bridging stent was performed. Previous publications report an applicability of the technique in various aortic pathologies, including fenestration creation and cheese-wire technique of the lamina in aortic dissections, and in situ endograft fenestrations.^7,11^

Balloon expandable covered stents (BECS) are widely used as bridging stents in f/bEVAR, with high TV patency, and without any significant outcome difference, when compared to self-expanding covered stents.^13^ Stent unmount may occur, especially in cases of demanding anatomy, as in high-grade stenosis, severe atheromatosis or due to friction within the introducer sheath and may escape attention, when the visualization of the BECS itself on fluoroscopy is restricted due to obesity and lateral projections. This misplacement could result in stent malposition, as in the current case. This could have been avoided either with the complete retraction of the stent before the performance of the angiography, the initial use of a larger sheath, which could permit the angiography despite the presence of the stent, or the retraction of the unmounted stent before deployment.

Stent misdeployment may lead to target or collateral vessel occlusion, such as the unintended CA coverage in this case. Some of these collaterals may play an important role in organ blood supply and preoperative detailed CTA evaluation is mandatory to detect adequate collateralization.^1^ Despite that intended overstenting of the CA in fEVAR and intentional occlusion of directional branches in bEVAR cases has been described, TV preservation should be attempted, whenever possible.^14^ Intentional CA coverage has been previously reported to relate to 16% severe complications, including visceral ischemia in 6% of cases, while may lead to death in almost 3% of these patients.^14^

Data arising mainly from cardiology cases showed that the electrified wire technique is a safe and effective approach, easily reproducible and cost-effective.^7^ In vascular surgery, the technique has been applied in venous and arterial revascularization procedures with encouraging outcomes.^8,9^ In the current case, the electrified wire technique permitted manipulations in an 8 mm PTFE-covered stent lumen and appropriate catheter position for fenestration creation. Other techniques would have not been feasible within this anatomy, as stiffer and larger diameter material may hamper gentle movements.^6^ The electrified wire technique seems to provide significant adaptability in demanding anatomic circumstances.

It is important to mention that before complete stent deployment, any attempt to cross the PTFE failed, potentially due to dense stent fabric. After full deployment, both balloon and catheter advancement were easily performed. Coronary balloons have been described previously for in situ fenestration creation to provide a graded dilation, while cutting balloons have been also applied. Conflicting outcomes exist as the current experience is limited, and various protocols are used in different centers, according to surgeons’ experience and preferences.^7^ After progressive dilatation, a balloon-expandable bare metal stent was deployed through the neo-fenestration creating a bifurcated bridging stent. Balloon-expandable stents permitted precise apposition and complete fenestration expansion.^6^

The presence of the bare metal stent across the visceral aorta did not affect the procedure, potentially due to the wide cells of the Sinus-XL.^15^ Previous experience showed that endovascular procedures are feasible in cases after PETTICOAT. However, several technical challenges could occur during the procedure mainly related to the wire entrapment between struts.^15^ A direct cause–effect correlation between the aneurysm development and the Sinus XL fracture cannot be confirmed. Five years after implantation, the PETTICOAT stent had multiple fractures. Sinus-XL (Optimed) is mainly used to address central venous system pathologies while data from patients with aortic disease are scarce, especially regarding dissection cases. Potential material fatigue could elaborate the presence of fracture and subsequently, aneurysm formation. Until further data are available, close follow-up is mandatory to detect material failure and indicate additional endovascular management.

## Conclusion

The electrified wire technique achieved fenestration creation within a bridging stent after early side branch occlusion. This technique can be used as a bailout solution in a wide range of aortic diseases and anatomies, with low cost, high effectiveness, and reproducibility.
